# Eukaryotic initiation factor-4E in superficial and muscle invasive bladder cancer and its correlation with vascular endothelial growth factor expression and tumour progression

**DOI:** 10.1054/bjoc.1999.0894

**Published:** 1999-12-08

**Authors:** J P Crew, S Fuggle, R Bicknell, D W Cranston, A de Benedetti, A L Harris

**Affiliations:** The Molecular Angiogenesis Group, Imperial Cancer Research Fund, The Institute of Molecular Medicine, Oxford, OX3 9DU, UK; The Nuffield Department of Surgery, The John Radcliffe Hospital, Oxford, OX3 9DU, UK; The Department of Urology, The Churchill Hospital, Oxford, OX3 7LJ, UK; The Department of Biochemistry and Molecular Biology, Louisiana State University Medical Centre, Shreveport, LA, 71130, USA

**Keywords:** angiogenesis, VEGF, eIF-4E, bladder cancer

## Abstract

Vascular endothelial growth factor (VEGF) is an important factor mediating tumour angiogenesis. VEGF mRNA is differentially expressed in bladder cancer with high expression in superficial tumours (stage pT_a_and pT_1_) contrasting with low expression in muscle invasive tumours (stage ≥ pT_2_). To investigate mechanisms regulating VEGF expression in bladder cancer, VEGF mRNA and protein were measured in normal bladder (*n* = 12) and primary bladder cancers (*n* = 57). VEGF protein levels correlated with mRNA expression in normal bladder (*r* = 0.68, *P* = 0.02) and bladder cancer (*r* = 0.46, *P* = 0.0007). Whilst VEGF mRNA expression was threefold higher in superficial compared to muscle invasive bladder cancers (*P* = 0.0001) there was no difference in VEGF protein (*P* = 0.81). Accordingly, the median protein:mRNA ratios increased more than 15-fold with increasing tumour stage (*P*< 0.0001) suggesting translational regulation. Expression of the eukaryotic initiation factor-4E (eIF-4E), a factor implicated in the translational regulation of VEGF, was greater in tumours than normal bladder (*P*< 0.0001) and correlated with VEGF protein:mRNA ratios (*n* = 43, *r* = 0.54, *P* = 0.0004) pointing to its role in the regulation of VEGF. In superficial tumours (*n* = 37) high expression of eIF-4E was associated with a poor prognosis and reduced stage progression-free survival (*P* = 0.04, Cox proportional hazards model). The study demonstrates that eIF-4E may be involved in translational regulation of VEGF in bladder cancer and might have a role as a prognostic factor in bladder cancer. © 2000 Cancer Research Campaign


*British Journal of Cancer* (2000) **82**(1), 161–166
© 2000 Cancer Research Campaign
Article no. bjoc.1999.0894

### Eukaryotic initiation factor-4E in superficial and muscle invasive bladder cancer and its correlation with vascular endothelial growth factor expression and tumour progression

**JP Crew[1,2,3], S Fuggle[2], R Bicknell[1], DW Cranston[3], A de Benedetti[4] and AL Harris[1]**

[1]The Molecular Angiogenesis Group, Imperial Cancer Research Fund, The Institute of Molecular Medicine, Oxford OX3 9DU, UK; [2]The Nuffield Department of Surgery, The John Radcliffe Hospital, Oxford, OX3 9DU, UK; [3]The Department of Urology, The Churchill Hospital, Oxford OX3 7LJ, UK; [4]The Department of Biochemistry and Molecular Biology, Louisiana State University Medical Centre, Shreveport, LA 71130, USA

**Summary** Vascular endothelial growth factor (VEGF) is an important factor mediating tumour angiogenesis. VEGF mRNA is differentially expressed in bladder cancer with high expression in superficial tumours (stage $pT_a$ and $pT_1$) contrasting with low expression in muscle invasive tumours (stage $\geq pT_2$). To investigate mechanisms regulating VEGF expression in bladder cancer, VEGF mRNA and protein were measured in normal bladder ($n = 12$) and primary bladder cancers ($n = 57$). VEGF protein levels correlated with mRNA expression in normal bladder ($r = 0.68$, $P = 0.02$) and bladder cancer ($r = 0.46$, $P = 0.0007$). Whilst VEGF mRNA expression was threefold higher in superficial compared to muscle invasive bladder cancers ($P = 0.0001$) there was no difference in VEGF protein ($P = 0.81$). Accordingly, the median protein:mRNA ratios increased more than 15-fold with increasing tumour stage ($P < 0.0001$) suggesting translational regulation. Expression of the eukaryotic initiation factor-4E (eIF-4E), a factor implicated in the translational regulation of VEGF, was greater in tumours than normal bladder ($P < 0.0001$) and correlated with VEGF protein:mRNA ratios ($n = 43$, $r = 0.54$, $P = 0.0004$) pointing to its role in the regulation of VEGF. In superficial tumours ($n = 37$) high expression of eIF-4E was associated with a poor prognosis and reduced stage progression-free survival ($P = 0.04$, Cox proportional hazards model). The study demonstrates that eIF-4E may be involved in translational regulation of VEGF in bladder cancer and might have a role as a prognostic factor in bladder cancer. © 2000 Cancer Research Campaign

**Keywords:** angiogenesis; VEGF; eIF-4E; bladder cancer

High vascular density is associated with a poor prognosis in muscle invasive bladder cancer (Dickinson et al, 1994; Bochner et al, 1995; Jaeger et al, 1995). The endothelial specific angiogenic factor, vascular endothelial growth factor (VEGF) is strongly implicated as a factor mediating this process with high VEGF expression related to a poor prognosis in a range of tumours including superficial bladder cancer (Crew et al, 1997). We previously demonstrated differential regulation of VEGF mRNA in bladder cancer with expression higher in superficial compared to muscle invasive tumours (O'Brien et al, 1995). Little is known regarding VEGF protein levels in these tumours although several mechanisms are involved in regulation of the VEGF gene, including a post-transcriptional component (Ferrara and Davis-Smyth, 1997). The eukaryotic initiation factor-4E (eIF-4E) is involved in the translational regulation of VEGF through increasing the efficiency with which the ribosome binds to mRNA (Rhoads, 1993; Kevil et al, 1996; Scott et al, 1997). The 7-methyl guanosine cap of mRNA is recognized by a cap-binding complex allowing unwinding of the 5′ untranslated region of the mRNA regulating attachment of the 40s ribosome and thereby facilitating translation. This process is the rate-limiting step in protein synthesis under normal conditions (Marcus, 1970; Darnbrough et al, 1973). The proto-oncogene eIF-4E is the least abundant factor within this complex and is itself rate-limiting for protein synthesis. In addition, eIF-4E can regulate cell growth (Jones et al, 1996), induce malignant transformation in cell lines (De Benedetti and Rhoads, 1990) and high expression has been demonstrated in tumours suggesting a role in tumour biology (Li et al, 1997; Nathan et al, 1997). Increased eIF-4E expression induces production of only a relatively small number of proteins (Rhoads, 1993). However, the poor prognosis associated with its high expression within a tumour may partly reflect regulation of factors involved in angiogenesis including VEGF (De Benedetti & Rhoads, 1990; Kevil et al, 1995; 1996).

The aim of this study was to quantify VEGF mRNA and protein in normal bladder and bladder cancers to determine their ratios in normal and neoplastic tissues. In addition, eIF-4E mRNA expression was quantified within the same cohort of tumours and correlated with the VEGF expression to establish its role in the regulation of VEGF in bladder cancer.

*Received 25 January 1999*
*Revised 30 June 1999*
*Accepted 5 July 1999*

*Correspondence to:* AL Harris, Institute of Molecular Medicine, University of Oxford, Oxford OX3 9DU, UK

### MATERIALS AND METHODS

### Patients and sample collection

Primary transitional cell carcinomas of the bladder were obtained from 57 patients undergoing transurethral bladder cancer resection

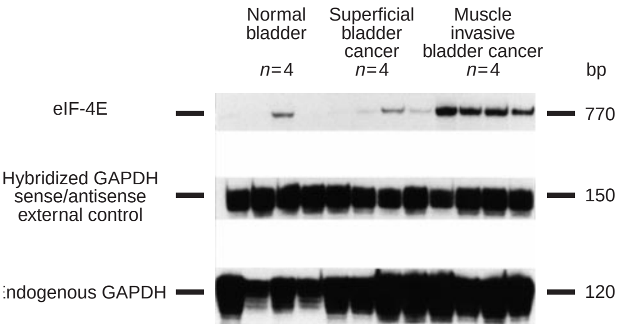

**Figure 1** A representative autoradiograph, following ribonuclease protection assay, illustrating high eIF-4E mRNA expression in muscle invasive transitional cell carcinomas of the bladder ($n$ = 4) compared to superficial transitional cell carcinomas of the bladder ($n$ = 4) or normal bladder samples (cadaveric organ donors, $n$ = 4). Expression was quantified using phosphorimager analysis and normalized to positive and external controls at The Churchill Hospital, Oxford, UK. Twelve specimens of macroscopically normal bladder were obtained from patients undergoing surgery for bladder cancer ($n$ = 5) or from cadaveric organ donors at the time of donor nephroureterectomy ($n$ = 7). All specimens were snap-frozen in liquid nitrogen at the time of resection.

### RNA and protein preparation

RNA was prepared according to the method of Chomczynski and Saachi (Chomczynski and Saachi, 1987). All RNA samples were run on a 1% agarose gel (under RNAase-free conditions) and the concentrations measured spectrophotometrically prior to ribonuclease protection analysis.

Tumour protein cytosolic and membrane fractions were prepared as previously described (Sacks et al, 1993). Briefly, tumours, frozen in liquid nitrogen, were morselized (cooled with liquid nitrogen) prior to homogenization, using a Dounce homogenizer, in 4 ml of HEPES–EDTA buffer containing proteinase inhibitors. This homogenate was centrifuged at 3000 ***g*** for 10 min (+4°C) and the supernatant further centrifuged at 100 000 ***g*** for 45 min (+2°C). The pellet was resuspended in 1 ml Tris-buffered saline (TBS) to form the ‘membrane’ fraction whilst the supernatant formed the ‘cytosol’ fraction. Total protein was quantified in all fractions using the Bradford method (Bradford, 1976).

### Ribonuclease protection assay

Two radiolabelled riboprobes were used to quantify VEGF and eIF-4E mRNA using ribonuclease protection. Plasmid pBluescript KS+ containing the full-length of VEGF was linearized with *Eco*RV and a 520-nucleotide antisense fragment was generated with T7 polymerase. Plasmid pGem-7Z containing the full-length of eIF-4E was linearized using ACC 1 and a 771 nucleotide antisense fragment generated with SP6 polymerase as previously described (De Benedetti et al, 1991).

The protected fragments for VEGF appeared as a doublet with the upper band (477 bp) corresponded to the VEGF 121 isoform whilst the lower band (427 bp) corresponded to the combined activity of the VEGF 165 and 189 isoforms. For the analysis the activity in both bands was summed (Crew et al, 1997).

In all protection assays an external control was used. This was formed from the hybridization of a sense and antisense riboprobe to glyceraldehyde-3-phosphate dehydrogenase (GAPDH) as previously described (Scott et al, 1997). Briefly, antisense and sense GAPDH riboprobes were generated (after linearizing the pBluescript KS+/GAPDH construct with *Hin*dIII and *Bam*H1 respectively) using T3 and T7 polymerase respectively.

Antisense riboprobes, labelled with [$^{32}$P]dCTP were hybridized to 10 μg of total cellular RNA, and the free unhybridized probe was digested with RNAase and T1. Following electrophoresis in a 6% polyacrylamide/urea-sequencing gel the protected fragments (Figure 1) were analysed using the ImageQuant, Phosphorimager (Version 3.3, Molecular Dynamics Inc., Sunnyvale, CA, USA). Analysis was performed in a blinded fashion prior to determining patient outcome.

To control against losses during the assay signals were normalized to the external control formed by hybridization of the sense and antisense GAPDH riboprobes (Crew et al, 1997). In addition, to allow accurate comparisons between ribonuclease protection assays a positive control was run on all electrophoresis gels. The positive control was given the value of 100 arbitrary mRNA units and all other signals were expressed relative to this. A breast cancer specimen known to express large quantities of eIF-4E mRNA was used as the positive control for eIF-4E (Scott et al, 1997) whilst a bladder cancer specimen known to express large quantities of VEGF mRNA was used as the positive control for VEGF (Crew et al, 1997).

### Enzyme-linked immunosorbent assay

The Quantikine™ enzyme-linked immunosorbent assay (ELISA) (R&D Systems, Abingdon, UK) was used to measure VEGF protein. Both membrane and cytosolic fractions were quantified in order to ascertain the site of VEGF protein and to determine whether extracellular sequestration and membrane binding of VEGF were significant. Fifty micrograms of total protein were assayed from each sample and the results expressed as ng VEGF mg$^{-1}$ total protein.

Prior to the study the ELISA was validated for use with tumour protein preparations. This was achieved by ensuring that the buffers used in the protein preparation did not interfere with the ELISA, and that VEGF retrieval from the preparations remained linear and parallel to the standard reference curve used in the ELISA.

### Statistics

All statistics were performed using Statview 4.5 (Abacus Concepts, Berkeley, CA, USA) or Stata 5.0 (Stata Corp, TX, USA).

### RESULTS

### VEGF protein expression in bladder cancer and normal bladder

The subcellular localization of VEGF protein expression within normal bladder ($n$ = 19) and bladder cancer ($n$ = 42, 26 superficial, 16 muscle invasive) was quantified in cytosolic and membrane fractions using an ELISA. There was no statistical difference between the concentration of VEGF in cytosolic or membrane fractions in any group (normal bladder $P$ = 0.68, superficial

*© 2000 Cancer Research Campaign*

**Table 1** Subcellular localization of VEGF protein in normal bladder and bladder cancer

| Tissue fraction | VEGF protein concentration (pg mg$^{-1}$ total protein) Normal bladder median (range) | Bladder cancer median (range) |
|---|---|---|
| Cytosolic | 42 (0–404) | 1643 (30–4902) |
| Membrane | 32 (0–238) | 1299 (128–6006) |

bladder cancer $P$ = 0.40, invasive bladder cancer $P$ = 0.07, Mann–Whitney $U$-test) although median VEGF expression was greater within the cytosolic compartment in all groups (Table 1). Since reconstitution of the cytosolic fraction was in a volume of 4 ml of buffer compared to only 1 ml for the membrane fraction the total VEGF protein content within the cytosolic compartment was greater than in the membrane compartment ($P$ < 0.01 in all groups). For the remaining analysis VEGF concentrations within the cytosolic fraction were used.

### VEGF mRNA and protein expression

RNA and protein preparations were quantified in 12 specimens of normal bladder and 57 bladder cancers (39 superficial tumours, 18 muscle invasive tumours). VEGF mRNA varied over 100-fold across the tumours. VEGF mRNA in both superficial tumours (median = 100, range 6–345, $P$ < 0.0001 Mann–Whitney $U$-test) and muscle invasive tumours (median = 30, range 7–67, $P$ = 0.06) were higher than in normal bladder (median = 9.5, range 1–78) (Table 2). In addition, levels were threefold higher in superficial tumours (median = 100 [standardized to the positive control], range 6–345) compared to muscle invasive tumours (median = 30, range 2–70, $P$ = 0.0001).

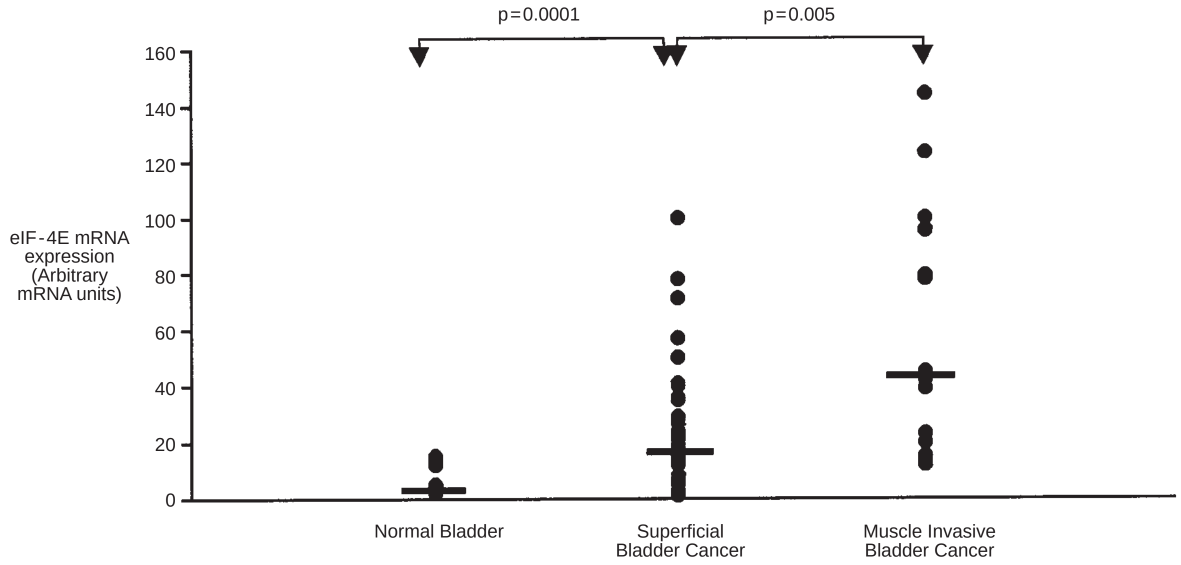

**Figure 2** EIF-4E mRNA expression derived from ribonuclease protection assay in normal bladder, superficial and muscle invasive bladder tumours. The figures represent expression relative to a positive control given the arbitrary value of 100 mRNA units. Median values are illustrated (bars) and $P$-values were generated using the Mann–Whitney $U$-test

**Table 2** Median and range of expression of VEGF mRNA, cytosolic protein and protein:mRNA ratios together with the correlating eIF-4E mRNA expression in normal bladder, superficial and muscle invasive bladder cancer

| | VEGF mRNA (arbitrary mRNA units) | | VEGF protein (pg mg$^{-1}$ total protein) | | VEGF protein:mRNA (pg mg$^{-1}$ total protein/ arbitrary mRNA unit) | | EIF-4E mRNA (arbitrary mRNA units) | |
|---|---|---|---|---|---|---|---|---|
| | **Median (range) *n* =** | ***P*** | **Median (range) *n* =** | ***P*** | **Median (range) *n* =** | ***P*** | **Median (range) *n* =** | ***P*** |
| Normal bladder | 9.5 (1–78) 12 | 0.0004 | 36 (1–326) 12 | <0.0001 | 4.0 (0.2–5.9) 12 | <0.0001 | 5 (2–15) 12 | <0.0001 |
| Bladder cancer | 57 (6–345) 57 | | 1477 (42–5000) 57 | | 21.1 (0.8–228.4) 57 | | 23 (1–144) 53 | |
| Superficial bladder cancer | 100 (6–345) 39 | 0.0001 | 1478 (42–5000) 39 | 0.81 | 14.2 (0.8–140.8) 39 | <0.0001 | 19 (1–100) 37 | 0.005 |
| Muscle invasive bladder cancer | 30 (7–67) 18 | | 1454 (48–4522) 18 | | 68.6 (17–228.4) 18 | | 44 (12–144) 16 | |

© 2000 Cancer Research Campaign

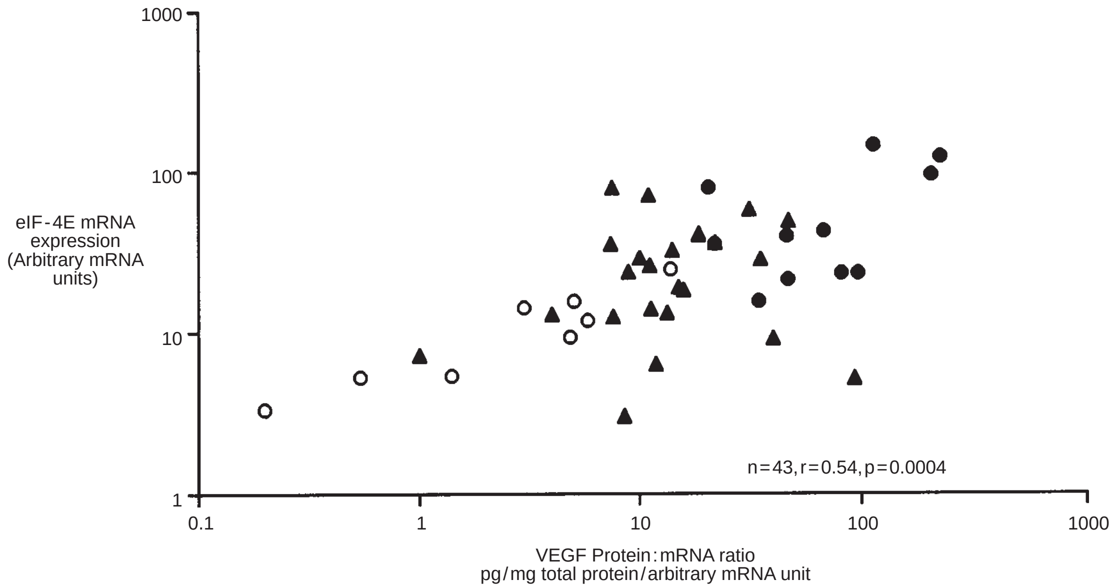

**Figure 3** The correlation between eIF-4E mRNA expression and VEGF protein:mRNA ratios in normal bladder ($n$ = 8, $r$ = 0.88, $P$ = 0.02, Spearmann's rank correlation), superficial and muscle invasive bladder cancer ($n$ = 35, $r$ = 0.36, $P$ = 0.03). Both axes are on logarithmic scales.
EIF-4E and VEGF mRNA are expressed as arbitrary mRNA units generated by normalization to a positive control as described in the text

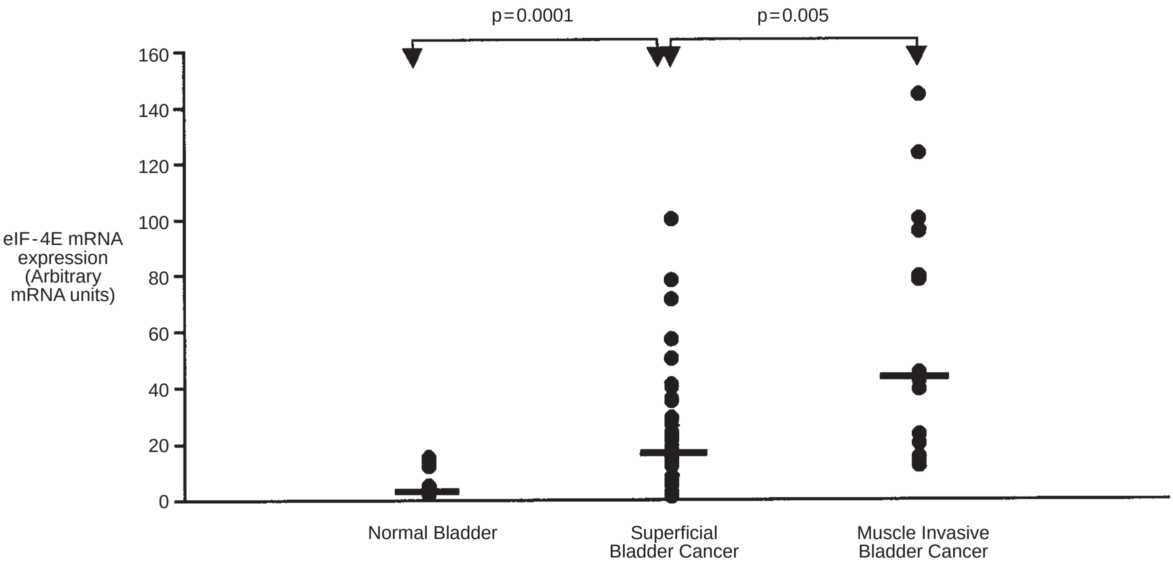

**Figure 4** Kaplan–Meier survival curves illustrating stage progression-free survival (**A**) and relapse-free survival (**B**) in 32 T1G1 and T1G2 bladder cancers expressing high eIF-4E mRNA ($n$ = 16 above the median) and low eIF-4E mRNA ($n$ = 16 below the median). Time to stage progression was shorter in tumours expressing high eIF-4E mRNA ($P$ = 0.04, Cox proportional hazards model). No difference was demonstrated in time to relapse ($P$ = 0.08), although levels were higher in tumours recurring by 6 months ($P$ = 0.01, $\chi^2$ test)

Levels of VEGF cytosolic protein varied over 100-fold across the tumours. There was no difference between VEGF protein levels in superficial (median = 1478 pg mg$^{-1}$ total protein, range 42–5000) or muscle invasive tumours (median = 1454, range 48–4522, $P$ = 0.81), although expression in both phenotypes was 40-fold greater than in normal bladder (median = 36, range 1–326, $P$ < 0.0001) (Table 2).

### The relationship between VEGF mRNA and protein expression

VEGF mRNA and protein expression correlated in both the normal bladder ($n$ = 12, $r$ = 0.68, $P$ = 0.02) and bladder cancer ($n$ = 57, $r$ = 0.46, $P$ = 0.0007). This correlation was more significant in superficial ($n$ = 39, $r$ = 0.58, $P$ = 0.0003) compared to muscle invasive tumours ($n$ = 18, $r$ = 0.48, $P$ = 0.05). Median VEGF protein:mRNA ratios was fivefold greater in the tumours compared to normal bladder ($P$ < 0.0001, Mann–Whitney $U$-test) indicating that more VEGF protein was generated per unit of mRNA in bladder cancer compared to normal bladder (Table 2). In invasive tumours low VEGF mRNA and high protein expression resulted in VEGF protein:mRNA ratios (median = 68.6, range 17–228.4) fivefold greater than in superficial tumours (median = 14.1, range 0.8–140.8, $P$ < 0.0001) (Table 2). These findings suggested that in these tumours VEGF was regulated by post-transcriptional mechanisms.

 © 2000 Cancer Research Campaign

### EIF-4E in translational regulation of VEGF in bladder cancer

Expression of eIF-4E mRNA was quantified in 12 normal bladder samples, 37 superficial (three stage Ta, 34 stage T1) and 16 muscle invasive bladder tumours using ribonuclease protection analysis. Expression was lower in normal bladder samples compared to bladder cancers ($P < 0.0001$, Mann–Whitney *U*-test) (Table 2). Figure 1 is a representative autoradiograph following ribonuclease protection illustrating the increased in eIF-4E mRNA expression in malignant compared to non-malignant bladder (Figure 1). Quantification of this signal following ribonuclease protection was achieved using phosphorimager analysis as described above. In bladder cancers there was a 144-fold variation in expression with levels higher in muscle-invasive compared to superficial tumours ($P = 0.005$, Mann–Whitney *U*-test) (Figure 2). The increase in eIF-4E mRNA expression correlated with increasing VEGF protein:mRNA ratios ($n = 43$, $r = 0.54$, $P = 0.0004$) (Figure 3). This correlation was present in both the normal bladder ($n = 8$, $r = 0.88$, $P = 0.02$) and bladder cancer ($n = 35$, $r = 0.36$, $P = 0.03$) indicating an association between the two factors, with eIF-4E involved in the regulation of VEGF translation in these tumours.

Poorly differentiated (G3) stage T1 tumour carry a poor prognosis with a high risk of stage progression. In this group of tumours ($n = 4$, median = 71, range 35–100) eIF-4E mRNA expression was significantly higher than in well (G1) or moderately (G2) differentiated T1 bladder tumours ($n = 32$, median = 18, range 1–78, $P = 0.01$). In T1G1 and T1G2 tumours ($n = 32$) eIF-4E expression above the median was associated, in univariate analysis, with reduced stage progression-free survival ($P = 0.04$, Cox proportional hazards model) (Figure 4) and an increased risk of recurrence by 6 months ($P = 0.01$, $\chi^2$ test) demonstrating that eIF-4E is a prognostic factor in these tumours. There was an association with survival in muscle invasive tumours ($P = 0.15$, Cox proportional hazards model).

### DISCUSSION

It is becoming increasingly apparent that VEGF is central to tumour angiogenesis and that high expression of VEGF is associated with a poor prognosis in some tumours. We have shown previously that high VEGF mRNA expression is associated with early recurrence and stage progression to a muscle invasive phenotype in superficial bladder cancer (Crew et al, 1997). In this study VEGF mRNA and protein were measured in 12 samples of normal bladder and 57 bladder tumours. Since VEGF protein levels were high in both superficial and muscle invasive tumour phenotypes, VEGF may still be important in determining the angiogenic properties within muscle invasive tumours despite the relatively low VEGF mRNA expression. Nevertheless, our previous hypothesis that superficial and muscle invasive tumours utilize different angiogenic pathways to stimulate the neovascularization (O'Brien et al, 1995) is still valid with post-transcriptional mechanisms regulating VEGF in muscle invasive tumour. Anti-VEGF therapeutic strategies have been postulated as new treatment options for superficial tumours (Crew et al, 1997) and this finding indicates that they may equally have a role in muscle invasive tumours.

Whilst VEGF mRNA and protein correlated in both superficial and muscle invasive tumours, protein:mRNA ratio were 17-fold higher in the latter phenotype compared to normal bladder and fivefold higher compared to the superficial phenotypes. This suggests translational regulation. The eIF-4E is implicated in the control of angiogenesis (Kevil et al, 1995) in part through regulation of VEGF protein translation. Chinese hamster ovary cells over-expressing eIF-4E have increased secretion of active VEGF over control cells with no difference in the transcript suggesting regulation of protein translation (Kevil et al, 1996). The importance of this factor in tumour biology and angiogenesis is further supported by increased expression in breast tumours compared to normal breast and in cell lines (Kerekatte et al, 1995; Anthony et al, 1996; Scott et al, 1997). Our study strengthens this postulate with median eIF-4E mRNA expression ninefold greater in muscle invasive bladder tumours compared to normal bladder and increasing with worsening tumour stage. The relationship between eIF-4E expression and tumour aggression may, in part, be mediated through the induction of VEGF and angiogenesis.

Whilst eIF-4E mRNA correlated with VEGF protein:mRNA ratios in all samples this correlation was poorer in malignant compared to normal bladder suggesting that factors other than eIF-4E may be involved in VEGF regulation within tumours. The regulation of both factors by a third factor, such as the c-*myc* gene (Rosenwald et al, 1993) could reduce the correlation and other members of the initiation complex could be dysregulated besides eIF-4E (e.g. eIF-4G). Binding of VEGF protein to the extracellular matrix could account for high VEGF protein:mRNA ratios, as previously described for other heparin binding angiogenic factor such as basic FGF (O'Brien et al, 1997). However, this hypothesis is not supported by our observations as no difference was found in the distribution of VEGF between membrane and cytosol fractions for different tumour stages. Furthermore, immunohistochemistry and in situ hybridization studies (manuscript in preparation) have indicated that it is the bladder cancer cells that express VEGF at both the protein and RNA levels respectively, suggesting that VEGF protein:mRNA ratios are intrinsic to the cancer cells rather than due to extracellular pools.

The study emphasizes the emerging importance of post-transcriptional mechanisms and eIF-4E in cancer biology and the pathogenesis of bladder cancer. Stage T1G1 and T1G2 bladder cancers form a highly heterogeneous group of tumours with a high risk of recurrence and stage progression carrying with it a poor prognosis. The prognostic factors presently available in this group of tumours are inadequate and eIF-4E could offer additional prognostic information. Since the correlation coefficient between eIF-4E and VEGF was 0.54 this indicates that this factor may give independent prognostic information compared to VEGF. Such a prognostic factor (or combination of factors) may be of clinical value allowing treatment or follow-up of bladder cancer to be tailored to individual patients.

### ACKNOWLEDGEMENTS

Jeremy Crew was supported by the Lillian May Coleman Research Scholarship of the Royal College of Surgeons of England. Roy Bicknell and Adrian Harris were supported by the Imperial Cancer Research Fund.

### REFERENCES

Anthony B, Carter P and De Benedetti A (1996) Overexpression of the proto-oncogene/translation factor 4e in breast carcinoma cell lines. *Int J Cancer* **65**: 858–863

Bochner BH, Cote RJ, Groshen S, Esrig D, Freeman JA, Weidner N, Chen S-C, Skinner DG and Nichols PW (1995) Angiogenesis in bladder cancer: relationship between microvessel density and tumour prognosis. *J Natl Cancer Inst* **87**: 1603–1612

Bradford MM (1976) A rapid and sensitive method for the quantitation of microgram quantities of protein utilizing the principle of protein dye binding. *Anal Biochem* **72**: 248–254

Chomczynski P and Saachi N (1987) Single-step method of RNA isolation by acid guanidium thiocyanate-phenol-chloroform extraction. *Anal Biochem* **162**: 156–159

Crew JP, O'Brien TS, Bradburn M, Fuggle S, Bicknell R, Cranston D and Harris AL (1997) Vascular endothelial growth factor is a predictor of relapse and stage progression in superficial bladder cancer. *Cancer Res* **57**: 5281–5285

Darnbrough C, Legon S, Hunt T and Jackson RJ (1973) Initiation of protein synthesis: evidence for messenger RNA-independent binding of methionyl-transfer RNA to the 40 S ribosomal subunit. *J Mol Biol* **76**: 379–403

De Benedetti A and Rhoads RE (1990) Overexpression of eukaryotic protein synthesis initiation factor 4E in HeLa cells results in aberrant growth and morphology. *Proc Natl Acad Sci USA* **87**: 8212–8216

De Benedetti AS, Joshi-Barve S, Rinker-Schaeffer C and Rhoads RE (1991) Expression of antisense RNA against initiation factor eIF-4E mRNA in HeLa cells results in lengthened cell division times, diminished translation rates, and reduced levels of both eIF-4E and the p220 component of eIF-4. *Mol Cell Biol* **11**: 5435–5445

Dickinson AJ, Fox SB, Persad RA, Hollyer J, Sibley GN and Harris AL (1994) Quantification of angiogenesis as an independent predictor of prognosis in invasive bladder carcinomas. *Br J Urol* **74**: 762–766

Ferrara N and Davis-Smyth TD (1997) The biology of vascular endothelial growth factor. *Endocr Rev* **10**: 4–25

Jaeger T, Weidner N, Chew K, Moore D, Kerschmann R, Waldmann F and Carroll P (1995) Tumour angiogenesis correlates with lymph node metastases in invasive bladder cancer. *J Urol* **154**: 69–71

Jones R, Branda J, Johnston K, Polmenis M, Gadd M, Rustgi A, Callanan L and Schmidt E (1996) An essential E box in the promotor of the gene encoding the mRNA cap-binding protein (eukaryotic initiation factor 4E) is a target for activation by c-myc. *Mol Cell Biol* **16**: 4754–4764

Kerekatte V, Smiley K, Hu B, Smith A, Gelder F and De Benedetti A (1995) The proto-oncogene/translation factor eIF-4E: a survey of its expression in breast carcinomas. *Int J Cancer* **64**: 27–31

Kevil C, Carter P, Hu B and De Benedetti A (1995) Translational enhancement of FGF-2 by eIF-4 factors, and alternate utilization of CUG and AUG codons for translation initiation. *Oncogene* **11**: 2339–2348

Kevil CG, De Benedetti A, Payne DK, Coe LL, Laroux FS and Alexander JS (1996) Translational regulation of vascular permeability factor by eukaryotic initiation factor 4E: implications for tumour angiogenesis. *Int J Cancer* **65**: 785–790

Li BD, Liu L, Dawson M and De Benedetti A (1997) Overexpression of eukaryotic initiation factor 4E (eIF-4E) in breast carcinoma. *Cancer* **79**: 2385–2390

Marcus A (1970) Tobacco mosaic virus ribonucleic acid-dependent amino acid incorporation in a wheat embryo system. Analysis of the rate limiting reaction. *J Biol Chem* **245**: 955–961

Nathan CA, Liu L, Li BD, Abreo FW, Nandy I and De Benedetti A (1997) Detection of the proto-oncogene eIF-4E in surgical margins may predict recurrence in head and neck cancer. *Oncogene* **15**: 579–584

O'Brien TS, Cranston D, Fuggle S, Bicknell R and Harris AL (1995) Different angiogenic pathways characterize superficial and invasive bladder cancer. *Cancer Res* **55**: 510–513

O'Brien TS, Cranston D, Fuggle S, Bicknell R and Harris AL (1997) Two mechanisms of basic fibroblast growth factor induced angiogenesis in bladder cancer. *Cancer Res* **57**: 136–140

Rhoads RE (1993) Regulation of eukaryotic protein synthesis by initiation factors. *J Biol Chem* **268**: 3017–3020

Rosenwald IB, Rhoads DB, Callana LD, Isselbacher KJ and Schmidt EV (1993) Increased expression of the eukaryotic translation initiation factor eIF-4E and eIF-2A in response to growth induction by c-*myc*. *Proc Natl Acad Sci* **90**: 6175–6178

Sacks NPM, Smith K, Norman AP, Greenall M, Lejeune S and Harris AL (1993) Cathepsin D levels in primary breast cancer: relationship with epidermal growth factor receptor, oestrogen receptor and axillary node status. *Eur J Cancer* **29A**: 426–428

Scott PAE, Smith K, Bicknell R and Harris AL (1997) A reliable external control for ribonuclease protection assays. *Nucleic Acids Res* **25**: 1305–1306

Scott PAE, Smith K, Poulsom R, De Benedetti A, Bicknell R and Harris AL (1998) Differential expression of vascular endothelial growth factor mRNA versus protein isoforms expression in human breast cancer and relationship to eIF-4e. *Br J Cancer* **77**: 2120–2128

*© 2000 Cancer Research Campaign*
